# Immune response profiles from humans experimentally exposed to *Phlebotomus duboscqi* bites

**DOI:** 10.3389/fimmu.2024.1335307

**Published:** 2024-04-03

**Authors:** Fernanda Fortes de Araujo, Maha Abdeladhim, Clarissa Teixeira, Kelly Hummer, Matthew D. Wilkerson, Roseanne Ressner, Ines Lakhal-Naouar, Michael W. Ellis, Claudio Meneses, Saule Nurmukhambetova, Regis Gomes, W. David Tolbert, George W. Turiansky, Marzena Pazgier, Fabiano Oliveira, Jesus G. Valenzuela, Shaden Kamhawi, Naomi Aronson

**Affiliations:** ^1^ Infectious Disease Division, Department of Medicine, Uniformed Services University of the Health Sciences, Bethesda, MD, United States; ^2^ Henry M Jackson Foundation for the Advancement of Military Medicine, Bethesda, MD, United States; ^3^ Vector Molecular Biology Section, Laboratory of Malaria and Vector Research (LMVR), National Institutes of Allergy and Infectious Diseases, NIH, Rockville, MD, United States; ^4^ Department of Biotechnology, Laboratory of Immunoparasitology, Oswaldo Cruz Foundation, Eusébio, CE, Brazil; ^5^ Department of Anatomy, Physiology and Genetics, Uniformed Services University of the Health Sciences, Bethesda, MD, United States; ^6^ Center for Infectious Disease Research, Walter Reed Army Institute of Research, Silver Spring, MD, United States; ^7^ University of Toledo Medical Center, Toledo, OH, United States; ^8^ Department of Dermatology, Uniformed Services University of the Health Sciences, Bethesda, MD, United States

**Keywords:** *P. duboscqi*, saliva, antigen, vaccine, immune response

## Abstract

**Introduction:**

Cutaneous leishmaniasis is a neglected vector-borne parasitic disease prevalent in 92 countries with approximately one million new infections annually. Interactions between vector saliva and the human host alter the response to infection and outcome of disease.

**Methods:**

To characterize the human immunological responses developed against saliva of *Phlebotomus duboscqi*, a *Leishmania major (L. major)* vector, we repeatedly exposed the arms of 14 healthy U.S volunteers to uninfected *P. duboscqi* bites. Blood was collected a week after each exposure and used to assess total IgG antibodies against the proteins of *P. duboscqi* salivary gland homogenate (SGH) and the levels of IFN-gamma and IL-10 from peripheral blood mononuclear cells (PBMCs) stimulated with SGH or recombinant sand fly proteins. We analyzed skin punch biopsies of the human volunteer arms from the insect bite site and control skin site after multiple *P. duboscqi* exposures (four volunteers) using immunohistochemical staining.

**Results:**

A variety of immediate insect bite skin reactions were observed. Late skin reactions to insect bites were characterized by macular hyperpigmentation and/or erythematous papules. Hematoxylin and eosin staining showed moderate mononuclear skin infiltrate with eosinophils in those challenged recently (within 2 months), eosinophils were not seen in biopsies with recall challenge (6 month post bites). An increase in plasma antigen-specific IgG responses to SGH was observed over time. Western Blot results showed strong plasma reactivity to five *P. duboscqi* salivary proteins. Importantly, volunteers developed a cellular immunity characterized by the secretion of IFN-gamma upon PBMC stimulation with *P. duboscqi* SGH and recombinant antigens.

**Discussion:**

Our results demonstrate that humans mounted a local and systemic immune response against *P. duboscqi* salivary proteins. Specifically, PduM02/SP15-like and PduM73/adenosine deaminase recombinant salivary proteins triggered a Th1 type immune response that might be considered in future development of a potential *Leishmania* vaccine.

## Introduction

Leishmaniasis is a neglected tropical disease caused by *Leishmania* parasites. Parasites are transmitted to the host during blood-feeding by infected female sand flies, namely, the *Phlebotomus* species in the Old World (Asia, Africa and Europe) and the *Lutzomyia* species in the New World (Central and South America). The most commonly observed type of this disease is cutaneous leishmaniasis (CL), which results in chronic skin ulcers and permanent scars on the affected regions of the body. CL is prevalent in 92 countries with approximately one million infections annually (1). Therapeutic options are limited and despite numerous investigations, there are to date no available human vaccines for *Leishmania* (2).

Sand flies deposit salivary proteins into the skin to facilitate the blood meal (3). Immunogenic molecules are present in sand fly saliva, some of them elicit a pro-inflammatory response that consequently reduces parasite transmission (3). However, several other salivary substances have been shown to downregulate this immunity and facilitate parasite infection (4).

Co-inoculation of *Leishmania* parasite and salivary gland extract has been reported to increase the parasite burden and cause exacerbation of lesion development in naïve animals (5–9). However, immunization of animals with sand fly salivary proteins (10–12) or salivary gland homogenate (SGH) (9, 13, 14) or pre-exposure to uninfected bites resulted in a protective immune response against leishmaniasis (15–22). The main protective response is associated with salivary antigen-specific T cells producing IFN-γ (4, 16, 18, 20).

Anti-salivary proteins immunity appears to be important, considering that the parasite is unavoidably naturally inoculated along with salivary proteins into the bite site. Salivary antigens might be effective components of vaccines directed against vector bite-transmitted pathogens, all the more promising considering the natural boosting that can occur in endemic areas. Herein, we evaluated the immune response profile from humans experimentally exposed to *Phlebotomus duboscqi* bites to identify suitable candidates for assessment as human leishmaniasis vaccine antigens.

## Materials and methods

### Study population

Sixty-eight healthy US volunteers at Walter Reed Army Medical Center, Washington D.C. were screened in order to enroll volunteers that met protocol inclusion criteria. The inclusion criteria were healthy military healthcare beneficiaries between 18 and 50 years old, with plans to remain in local area for the next 12 months, and willingness to participate in all study procedures. The exclusion criteria included prior deployment/travel > a 30 day contiguous period to geographic areas where *Leishmania* transmitting phlebotomine sand flies are present (Southwest Asia/Sub-Saharan Africa), positive IgG antibody to sand fly saliva, pregnancy, elevated serum IgE >144 kU/L, history of chronic medical illness, large local reactions to insect bites, problems with prior phlebotomy or use of medications that may interfere with immune responses.

The study was approved by Institutional Review Boards of the Walter Reed Army Medical Center, the Uniformed Services University of the Health Sciences, and the National Institute of Allergy and Infectious Diseases (NIAID), (Protocol number WR355023). All clinical investigations were conducted according to the Declaration of Helsinki principles and all volunteers provided written informed consent. The study was registered on www.clinicaltrials.gov as NCT01289977.

**Figure 1:**
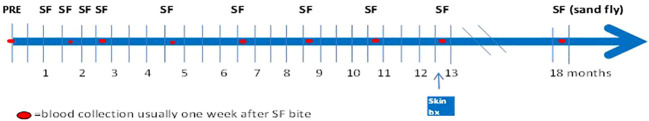
Timeline of Sand Fly Feeding (SFF) and blood collection. Healthy volunteers (n=14) were exposed to bites of uninfected *P. duboscqi* sand fly on a bi-weekly basis for the first two months and once every 2 months for the following 10 months, with an optional recall exposure 6 months after completion. Blood collections were performed to assess development of the specific humoral and cellular immune responses.

Participants had a medical history taken, which included a detailed allergic history, travel history, and physical examination. After screening, 14 volunteers were enrolled and their deltoid area exposed for 20 minutes to 10 colony-reared, uninfected *P. duboscqi* sand flies on a bi-weekly basis for the first two months and once every 2 months for the following 10 months, with an optional recall exposure 6 months after completion (**Figure 1**). Bite site skin reactions were observed for 10 minutes after the feeder was removed by study physicians; any volunteer with a large allergic reaction was observed for a longer period. Photographs were taken of the bite site and a description of the physical appearance as well as the presence of any symptoms was noted. Blood was collected from participants 7 ± 3 days following exposure to sand fly bites, when the bite site was also re-assessed. An optional substudy included a 3mm skin punch biopsy that was obtained from the bite site skin of the arm and a 2 mm control biopsy on the contralateral arm of each volunteer performed 48 hours after the final (8^th^ or 9^th^) *P. duboscqi* exposure (**Figure 1**).

### Exposure to colony-reared sand flies and SGH preparation

#### Human controlled exposure to uninfected laboratory-raised *P. duboscqi*

**Figure 2:**
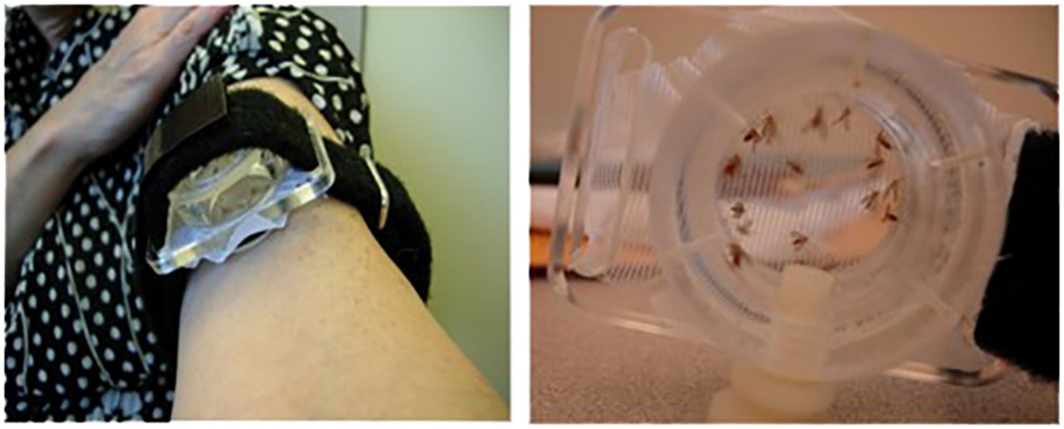
Demonstration of the sealed feeding apparatus. The feeding device containing ten (10) uninfected female sand flies was individually attached to the arm of each volunteer for 20 minutes.

Mali field-collected *P. duboscqi* sand flies were reared in the pathogen-free, state of the art insectary at the Laboratory of Malaria and Vector Research, NIAID (23). Sand flies were starved overnight. A feeding apparatus pre-filled with 10 female *P. duboscqi* sand flies was placed on the skin of the upper arm of volunteers, lightly covered, and sand flies left to feed for 20 minutes. The feeders are sealed plexiglass chambers that have a fine-mesh surface that permits the sand flies to feed (Precision Plastics Inc, Maryland USA) (**Figure 2**). Feeding sites were alternated between arms during subsequent visits. Several persons had tattoos on their arms, and avoidance of these areas was attempted. At the end of exposure all sand flies were examined under microscopy and an assessment of blood meal was performed. Participants were asked to refrain from using antihistamines or topical steroids for bite site symptoms, until consultation with study physicians.

#### Preparation of *P. duboscqi* salivary gland antigen

Salivary Gland Homogenate (SGH) was prepared by dissection of salivary glands from seven-day old laboratory-reared adult female *P. duboscqi*, submitted to ultra-sonication with a Branson Sonifier 450 for three 30 second cycles followed by centrifugation at 15,000 rpm for 3 minutes at 4°C. Supernatant was collected and stored at -80°C until use.

### Blood collection and storage

Blood was collected in heparinized tubes (BD Diagnostics, Hunt Valley, MD). Plasma was stored at -80°C. Peripheral Blood Mononuclear Cells (PBMCs) were isolated by density-gradient centrifugation using a Ficoll-Paque PLUS solution (GE Healthcare, Pittsburgh, PA). Cells were then counted and frozen in fetal bovine serum with 10% dimethyl sulfoxide solution (FBS-DMSO 10%) at -80°C overnight, before they were transferred to liquid nitrogen.

### Skin biopsies

Subjects could participate in an optional skin biopsy to assess the delayed hypersensitivity reaction at the site of the sand fly bites. This was performed 48 hours after the final sand fly biting exposure (Miltex sterile biopsy punch 3mm or 2mm). Skin tissue was then bisected with half placed in 10% formalin and half in RNA-later solution (Ambion). The formalin-treated skin biopsies underwent hematoxylin/eosin and immunohistochemical staining (Histoserv Inc, Germantown MD). Primary antibodies against CD3 (Dako#A0452, Carpinteria, CA) at a dilution of 1:100, CD4 (Dako#M7310) at 1:80, CD8 (Dako#M7103) at 1:75, CD20 (Dako#M0755) at 1:300, CD68 (Dako#M0814) at 1:100 and Myeloperoxidase (Dako#A0398) at 1:400 were used. For secondary antibodies biotinylated anti-mouse IgG against CD4, CD8, CD20, CD68, and biotinylated anti-rabbit IgG against CD3 at 1:500 dilution were used followed by streptavidin-horseradish peroxidase. Pictures were taken using an Olympus DP73 camera microscope BX51 and Cellsens Dimension Olympus software. Percentage of positive markers was identified qualitatively utilizing Image J software.

### Anti-SGH antibody detection by ELISA

Specific anti-*P. duboscqi* saliva IgG antibodies were assessed by ELISA. A 96-well high binding microtiter plate (Thermo Scientific, Rochester, NY) was coated with 50 μl of SGH diluted to one pair/ml in 0.1 M carbonate-bicarbonate buffer overnight at 4°C. The wells were then washed in Tris-buffered-saline (TBS) with 0.05% Tween 20 and incubated with TBS-4% Bovine Serum Albumin (BSA) for one hour at room temperature (RT) to block free binding sites. After three washes, 50 μl of 1:100 diluted sample plasma was added and incubated for 1 hour at 37°C. Antibody-antigen complexes were detected using alkaline phosphatase-conjugated goat anti-human IgG (H+L) antibodies (Sigma, MO) diluted at 1:5000 for 1 hour at RT and were visualized using nitrophenyl phosphate liquid substrate system (Sigma). The absorbance was measured at 405 nm using a Versamax microplate reader (Molecular devices).

### Anti-SGH antibody by Western Blot

Salivary glands (40 pairs, approximately equivalent to 40 mg total protein) were run on a 2D 4-12% NuPAGE gel (Novex Life Technologies). After transfer to a nitrocellulose membrane using the Transblot Turbo Transfer system (Biorad), the membrane was blocked with 5% nonfat dry milk in TBS-0.05% Tween pH 8.0 overnight at 4°C. After washing with TBS-T pH 8.0, the membrane was placed on a mini-protean II multiscreen apparatus (BioRad, Hercules, CA) and different lanes were incubated with various sera diluted 1:100 for 3 hours at RT. After washing with TBS-T pH 8.0 three times, the membrane was incubated with anti-human IgG (H+L) alkaline phosphate-conjugated antibody (1:5,000) (Sigma A1643) for 1 hour at RT. Membrane bands were developed with Thermo Scientific™1-Step™ NBT/BCIP substrate solution with the reaction stopped by washing the membrane with deionized water.

### Cloning and protein expression of *P. duboscqi* salivary proteins

DNA of the most abundant salivary molecules from *P. duboscqi* was amplified by polymerase chain reaction (PCR) using a forward primer deduced from the amino-terminus sequence (starting after the signal peptide) and a reverse primer encoding a hexa-histidine motif. The PCR conditions were: one hold for 5 minutes at 94°C, two cycles of 30 seconds at 94°C, 1 minute at 46°C, 1 minute at 72°C and 23 cycles of 30s at 94°C, 1 minute at 52°C, 1 min at 72°C and one hold of 7 min at 72°C. The PCR product was cloned into the VR2001- TOPO vector as previously described and then sequenced (24).

The VR-2010 plasmid coding for the target proteins containing a 6-histidine tag was expressed in HEK-293F cells. In short, the HEK-293F cells were grown in Expi293 medium (A1435101, ThermoFisher) at 37°C, 125 rpm, 8% CO_2_ atmosphere with 80% humidity in plastic flasks with ventilated caps (Corning^®^ Erlenmeyer sterile polycarbonate with 0.2 μm ventilated caps). One hundred μg of plasmid DNA was mixed with 200μL of PEI Max (24765–1, Polysciences) solution and 10mL of OPTI-MEM (Gibco) media and added to the cell culture and incubated under standard conditions for 3 hours. After incubation, the cells were transferred to a larger flask and diluted with pre-warmed Expi293 medium to a concentration of 1 mvc/ml. The transfected cells were incubated for 7 days at 37°C, 125 rpm, 8% CO2 atmosphere without handling them.

The codon optimized PduM73/adenosine deaminase sequence containing a 6-histidine tag was synthesized by Genscript and cloned into the pcDNA3.1 expression vector. PduM73 was then generated by transient transfection into 293F cells (ATCC). Briefly 50 µg of the expression plasmid was added to 5 ml of Opti-MEM reduced serum medium (Gibco) in one tube and 0.15 ml of sterile PEI (1 mg/ml in water) was added to separate 5 ml of Opti-MEM medium in another tube. The DNA was then filter sterilized and added dropwise to the PEI and the mixture incubated at room temperature for 30 minutes. DNA and PEI were then added to 30 ml of 293F cells (3-4x10 6 cells/ml) in Freestyle 293 expression medium. An additional 70 ml of Freestyle 293 expression medium was added to the cells three to six hours later. Cells were then grown at 37°C and 8% CO 2 for 6-7 days. At harvest cells were pelleted by centrifugation and the supernatant filtered through a 0.2 µm filter. PduM73 was purified from supernatant passed over a HisTrap HP nickel NTA column (Cytiva) equilibrated in 25 mM Tris-HCl pH 8.0 and 0.5 M sodium chloride. The column was washed with 25 mM Tris-HCl pH 8.0 and 0.5 M sodium chloride and PduM73 was eluted with 25 mM Tris-HCl and 0.1 M imidazole pH 8.0. Eluted protein was concentrated and then loaded on a Superdex 200 gel filtration column (Cytiva) equilibrated phosphate buffered saline (PBS) pH 7.2. An elution peak corresponding to a molecular weight of approximately 73 kDa was collected and concentrated for use in further experiments. Protein purity was assessed by SDS-PAGE.

### Cell culture

Human volunteer peripheral blood mononuclear cells (PBMCs) were cultured in RPMI 1640 medium supplemented with 10% fetal bovine serum (Sigma, St Louis, MO), 1 mM L-glutamine, 100 units/ml penicillin, 100ug/ml streptomycin. Frozen cells were removed from liquid nitrogen storage, thawed quickly at 37°C, and incubated with RPMI media with 0.1 mg/ml DNase for 15 minutes at RT. After incubation cells were centrifuged at 1300 rpm, 12°C and resuspended in RPMI media and left overnight in a 5% CO_2_ humidified atmosphere at 37°C. After incubation, cells were washed and counted (dead cells were excluded) using trypan blue solution (Hyclone, Thermo Scientific) and adjusted to 5x10^6^ cells/ml. One hundred μl of cells were then cultured in 96-well plates in cell culture medium with a 200ul final volume and incubated with SGH (2 pairs/ml) or 10 μg/ml of recombinant salivary *P. duboscqi* proteins (PduM02/SP15, PduM10/Yellow-SP44, PduM34/SP32, PduM35/Yellow-SP42, PduM49/SP12 and PduM73/adenosine deaminase) or phytohemagglutinin (PHA) (2% v/v) in a 5% CO_2_ humidified atmosphere at 37°C. Supernatants were collected after 96 hours, centrifuged, and stored at -80°C until use.

### IFN-gamma and IL-10 detection assay

For IFN-γ and IL-10 detection, supernatants of cell culture were collected after 96 hours, centrifuged at 1400 rpm at 4°C for 10 minutes and stored at -80°C until use. Following manufacturer instructions, supernatants were tested with human IFN-γ or IL-10 uncoated ELISA kits (Invitrogen). The results were interpolated from a standard curve using recombinant cytokines and expressed as concentration (pg/ml) of IFN-γ or IL−10 relative to non-stimulated cells.

### Statistical analysis

All tests were performed using GraphPad Prism 10 (GraphPad Software, Inc.). Wilcoxon test and one tailed t-test was used for comparison of IgG levels at each exposure with the pre-exposure (baseline). Mann Whitney test was used to compare selected cytokine differences between SGH and each recombinant protein and Wilcoxon signed rank test was used to compare PBMC culture results between stimuli and controls. Differences were considered significant if p value < 0.05.

## Results

### Characteristics of study volunteers

**Figure 3:**
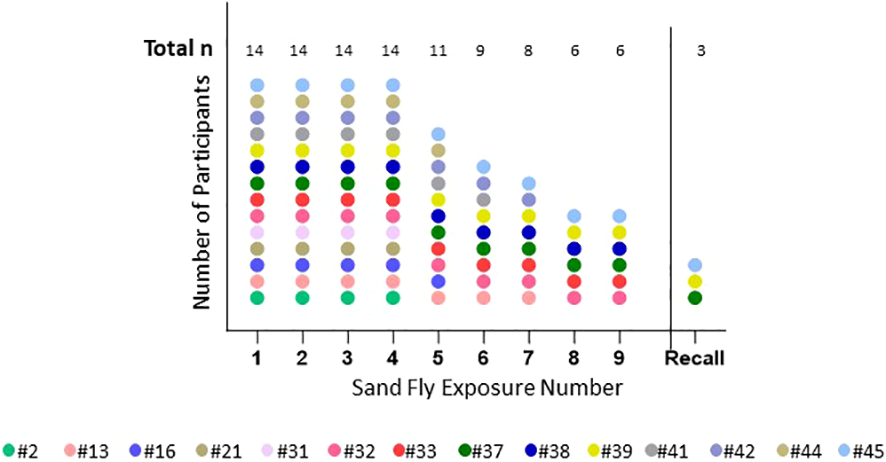
Number of completed sand fly exposures. This shows the number of *P. duboscqi* exposures that each participant completed. Recall means challenge after a 6 month hiatus post exposure. Each color represents one participant.

The median age of volunteers was 24.5 (range 21-50) years; the majority 9/14 (64.5%) were male and 12/14 (85.7%) of volunteers self-reported white race, one American Indian and one African-American. Demographics, travel and medical history of the cohort as well as the details of screening IgE levels are presented in **Table 1**. There was significant loss to follow up later in the trial (**Figure 3**), due mainly to site hospital closure where the majority of participants being active-duty military were reassigned/moved or left the service and were no longer eligible to be followed.

**Table 1:** Characteristics of study volunteers.

Subject number	Completed SF exposure	Age	Race	Sex	Prior travel	Allergic history	Concomitant medication	Baseline serum IgE (KU/L)
**2**	4	22	W	M	None	None	None	7.44
**13**	7	24	W	M	None	Seasonal allergies	None	22.2
**16**	5	21	W	F	None	Seasonal allergies Eczema	Oral Contraceptive	< 2
**21**	4	31	W	M	Kenya, Peru	None	Synthroid Omeprazole	41.2
**31**	4	23	W	M	Mexico	None	None	116
**32**	9	27	W	M	Honduras	Childhood asthma	Ranitidine Bupropion Sertraline	16.4
**33**	9	24	W	F	None	None	None	12.4
**37**	9	38	W	M	None	None	None	18.2
**38**	9	21	AA	M	None	None	None	129
**39**	10	25	W	F	Belize	Anaphylaxis to morphine Serous otitis media	None	4.02
**41**	6	50	W	M	Venezuela	None	Aspirin Ranitidine Simvastatin	36.9
**42**	7	37	W	F	Mexico	Seasonal allergies	Fluoxetine Esomeprazole	25.2
**44**	5	26	AI	M	None	Urticaria with amoxicillin	None	51.4
**45**	10	24	W	F	None	None	Methylphenidate	20.8

SF, sand fly; W, white; AA, African American; AI, American Indian; M, male and F, female.

One volunteer stopped intervention due to adverse events (immediate large wheal and flare allergic response) as was medically advised. Among participants, modest increases in serum IgE were seen over time after multiple exposures (data not shown). There was one protocol violation for participant #44 who received an exposure to *Lutzomyia longipalpis* instead of *P. duboscqi* on the fifth session, which was not recognized until after the completion of the feeding session; this participant had no further sand fly challenges.

### Human responses to *P. duboscqi* bites

Based on skin inspection immediately after the feeder was removed, all participants had at least one bite site noted each time. Assessment of sand flies post feeding showed that some had partial feeding, but majority had complete blood engorgement (**Supplementary Figure 1**). Every participant had at least one adverse event reported. Most were grade 1 related local reactions but there were ten grade 2 related local reactions. Unrelated grade 1 adverse events were upper respiratory tract infection (URI, 4), ankle sprain, conjunctivitis, headache (2), seasonal allergies, muscle soreness. Unrelated grade 2 adverse events were post phlebotomy hematoma, heartburn, URI, dysentery, acute gastroenteritis (2), myalgias. There were no grade 3 or 4 adverse events.

**Figure 4:**
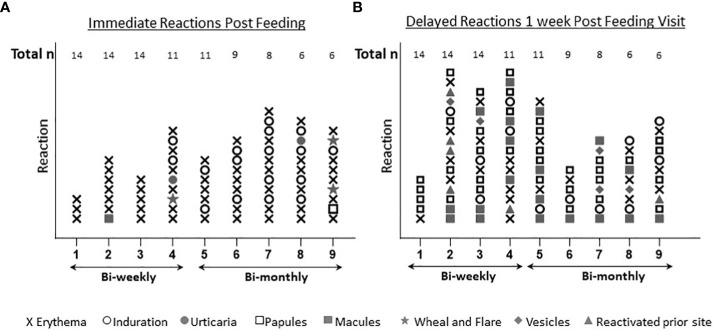
Immediate and delayed skin reactions post feeding. **(A)** Represents the number and type of skin reactions immediately after feeding for each exposure time. **(B)** Represents the number and type of skin reactions after one week post *P. duboscqi* challenge for each exposure time.

**Figure 5:**
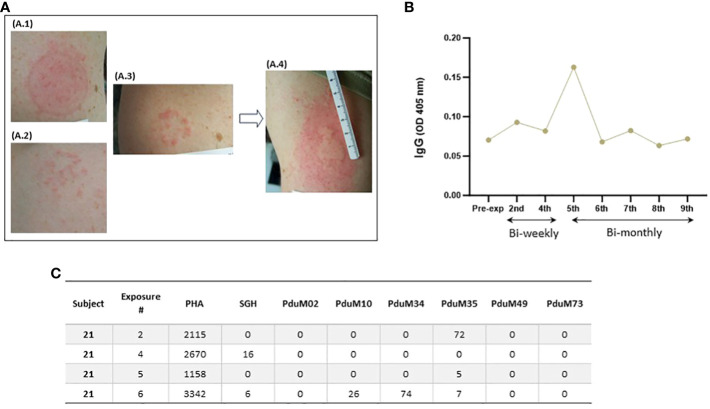
Serial immediate skin reactions and systemic immune responses after *P. duboscqi* feeding on participant #21 (removed from further sand fly challenge after 4^th^ exposure). **(A.1)** Participant arm at exposure 1; **(A.2)** Participant arm at exposure 2; **(A.3)** Participant arm immediately after removing feeder at exposure 4; **(A.4)** Participant arm 5 minutes after removing feeder at exposure 4; **(B)** Plasma levels of IgG antibodies to *P. duboscqi* saliva of participant at different time points. **(C)** IFN-gamma (pg/ml) production to *P. duboscqi* SGH and recombinant proteins at various timepoints.

Immediate skin reactions increased with the number of sand fly exposure sessions, whereas delayed skin reactions were more common during the initial phase of every two week sand fly exposures (**Figure 4**); however, by the time of exposure eight and nine, two participants presented with immediate wheal and flare (**Figure 4A**), and increasing delayed effects were noted (**Figure 4B**). Acute allergic responses did not correlate with high baseline IgE levels. An immediate wheal and flare reaction on exposure 4 led to termination of a participant #21 from further exposures (**Figure 5A**), although they continued to provide blood samples for the entire study allowing assessment of the kinetics of antibody and cellular responses over post exposure time. The IgG levels from participant #21 showed a peak of IgG levels after the 4^th^ exposure; subsequently the IgG levels decreased to baseline (**Figure 5B**). It is interesting that this participant had traveled to Kenya previously (but met inclusion criteria as it was less than 30 days) and could have been pre-exposed to *Phlebotomus* species including *P. duboscqi* in Africa. In addition, this participants’ PBMCs showed a weak and inconsistent cellular response to stimulation with *P. duboscqi* SGH and recombinant proteins in cell supernatants months after exposure termination (exposure 4) (**Figure 5C**).

**Figure 6:**
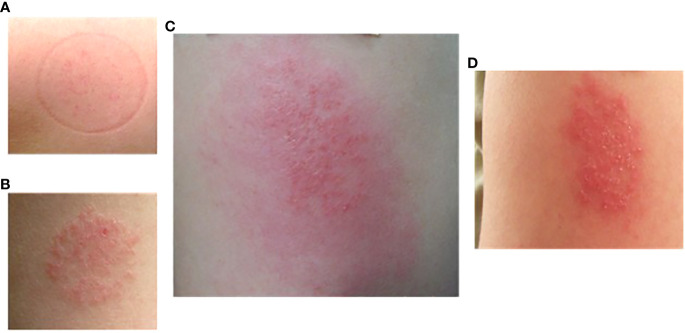
Delayed large local reaction from participant #16 after two exposures (different arms) of *P. duboscqi* bites. **(A)** Participant arm at exposure 1; **(B)** Participant arm at exposure 2; **(C)** Participant arm 2 days after exposure 2; **(D)** Participant arm 7 days after exposure 2.

Delayed skin reactions (often starting 2-3 days after exposure) could be quite remarkable, (**Figure 6**) but the same individuals would tolerate repeat exposure (contralateral arm) and often not demonstrate the same effect. Interestingly, intermittently participants reported late reactivation to prior bite sites with contralateral arm challenge, generally pruritic papules with or without erythema.

**Figure 7:**
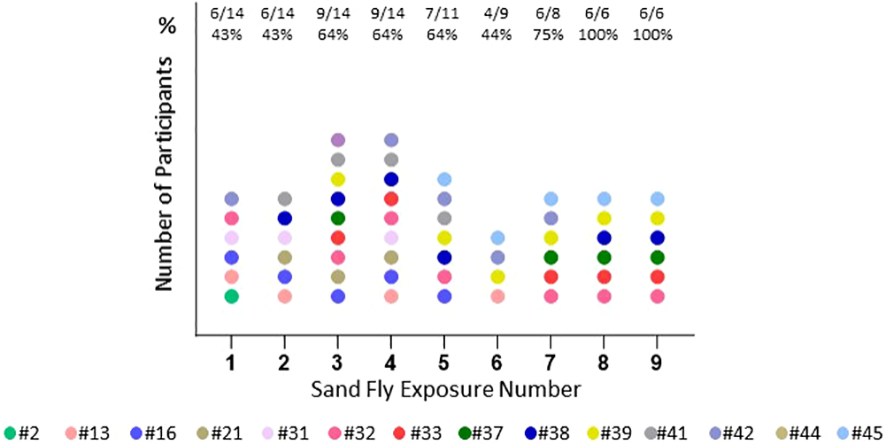
Pruritus reported by participant. Number and percentage (%) of participants with pruritus at each exposure time. Each color represents one participant (n=14).

Pruritus was a commonly reported symptom increasing in frequency over time (**Figure 7**). Pruritus was immediate in onset post exposure or delayed and usually had a duration of days, infrequently more than a week.

### Skin immune responses to *P. duboscqi* bites

#### Skin biopsy

**Figure 8:**
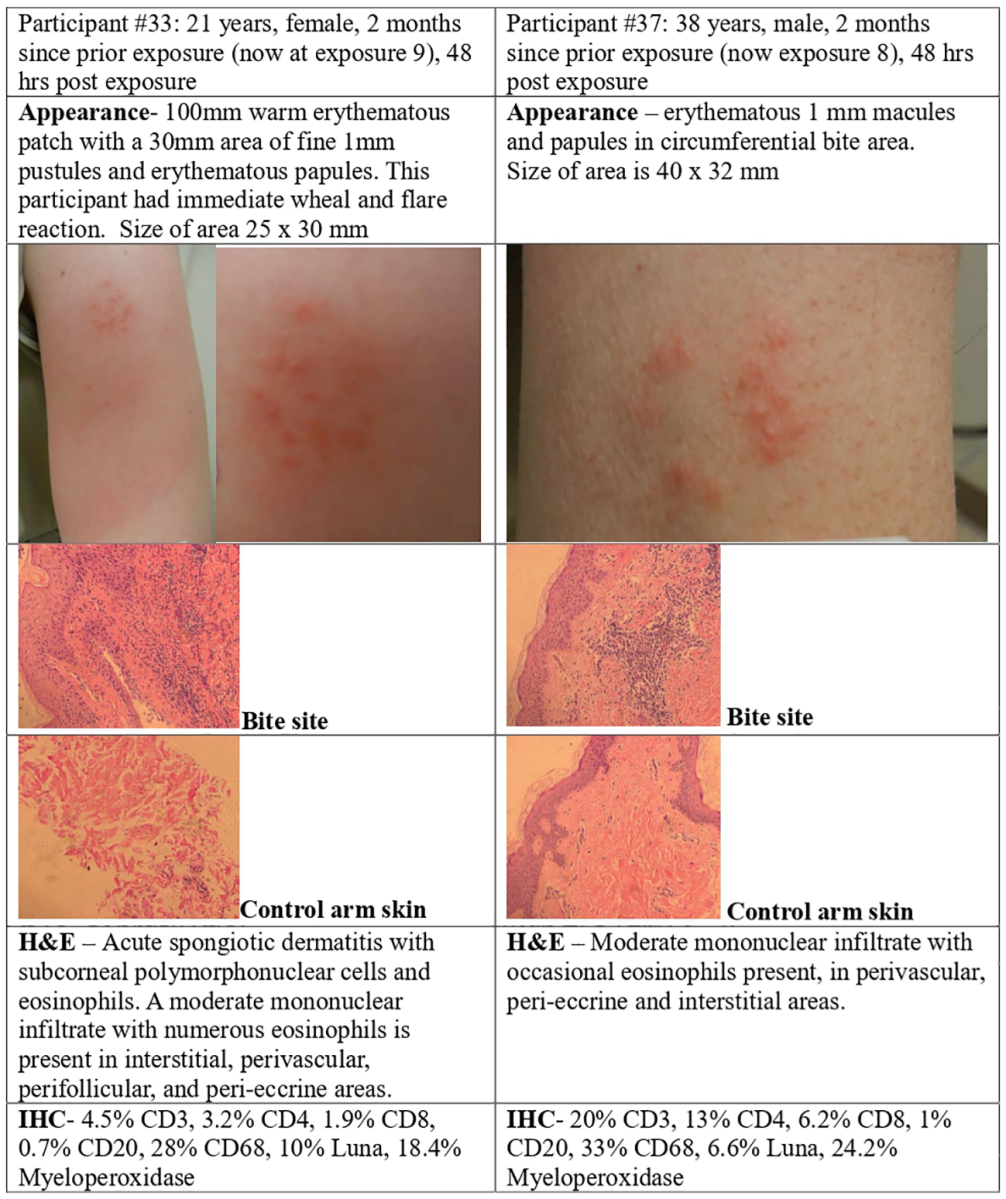
*P. duboscqi* challenge, 48 hour assessment including skin biopsy. Cellular infiltrate characteristics and bite site appearance from two participants that received the previous *P. duboscqi* exposure 2 months prior (now at exposures 8 and 9), hematoxylin/eosin staining (H&E) and immunohistochemical (IHC) staining.

**Figure 9:**
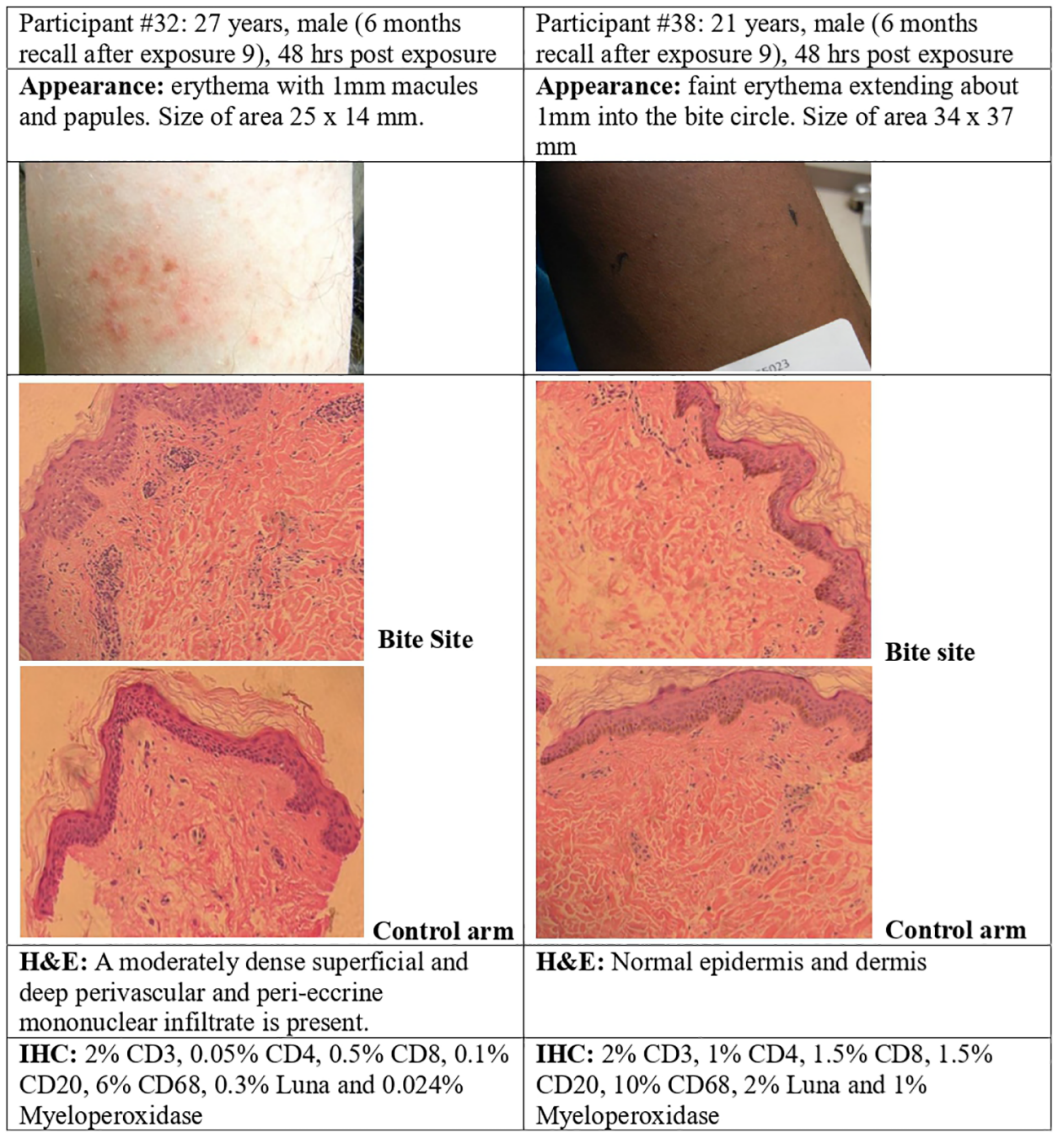
*P. duboscqi* challenge, 48 hour assessment including skin biopsy. Skin biopsy characteristics and bite site appearance from two participants that had received the previous *P. duboscqi* exposure 6 months prior, hematoxylin/eosin staining (H&E) and immunohistochemical (IHC) staining.

Punch skin biopsies of the bite site were obtained 48 hours after *P. duboscqi* exposure (**Figure 1**). **Figures 8**, **9** show results from four volunteers; two had received the previous *P. duboscqi* exposure 2 months prior to the biopsy (**Figure 8**) and two had received the previous *P. duboscqi* exposure 6 months prior (to assess immune recall responses) (**Figure 9**). The two participants who had skin biopsies while still on every 2-month *P. duboscqi* exposure schedule had more acute site inflammation than those volunteers with a preceding six month hiatus of exposure. One had an acute wheal and flare immediately post feeding and a large warm, tender, erythematous area with papules and pustules at the feeding site after 48 hours observation. Biopsy showed acute spongiotic dermatitis with neutrophils and numerous eosinophils (**Figure 8**). While this response occurred in the sole female who underwent a skin biopsy, wheal and flare responses were also seen in males in the study who did not undergo skin biopsy. Both participants on the 2 month challenge schedule had skin biopsies with eosinophils, and a moderate mononuclear infiltrate present. Immunohistochemistry showed lymphocytes and macrophage/monocytes, a few CD4, CD8, and positive Luna (eosinophils, mast cells) and Myeloperoxidase (neutrophils) staining. This was similar between both participants, although it differed in severity. Additionally, biopsies of control skin from the contralateral control arm were interpreted as normal skin, except in the female where mild perifollicular mononuclear inflammatory changes were seen in one section, albeit the area biopsied had likely been used for bite site in the past.

The recall exposure (6 months *P. duboscqi* exposure) skin responses differed from the every two month exposures. The sole African American participant (skin biopsy after 6 months, recall exposure) showed minimal skin reaction after each feeding and this was confirmed by routine histopathology interpreted as normal skin (the biopsy was taken within the circle of the feeder). Immunohistochemistry suggested a low level of lymphocyte and mononuclear cell staining (**Figure 9**). The nature of cellular infiltration was similar to the other participant with recall feeding, although this skin showed more inflammatory changes; at 48 hours after the bite session where the site had papules and erythema, histology demonstrated a dense mononuclear infiltration, and the cell types were mainly lymphocytes and macrophage/mononuclear cells, a few T cells, and a few B cells, with very low staining for eosinophils and neutrophils (**Figure 9**).

### Systemic immune responses developed to *P. duboscqi* saliva

#### Total IgG antibody response

**Figure 10:**
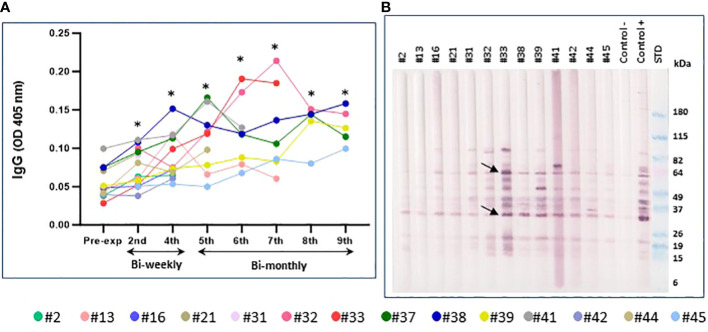
Immunoreactivity to Pd salivary protein. **(A)** Plasma levels of IgG antibodies to *P. duboscqi* saliva in experimentally exposed human volunteers (n=14) at different time points. Pre-exposure is before sand fly bite exposure. The * represents the statistical significance between the time points when compared to pre-exposure. **(B)** Western blot of sand fly salivary proteins recognized by IgG (diluted 1:5000) from plasma (diluted 1:100) of volunteers exposed to the bite of *P. duboscqi*. One donor from the NIH blood bank was used as a negative control and one donor from a Mali endemic area was used as a positive control. Arrows represent some immunodominant proteins (~ 62 and 32kDa).

The total IgG antibody response was evaluated in the volunteers exposed to SF bites during the time points studied, up to a maximum of nine exposures, approximately a week after *P. duboscqi* exposure. Our results demonstrated a low level of total IgG; however, a modest increase in plasma antigen-specific IgG response to SF salivary proteins was seen over time. Significant statistical differences were observed from all the exposure times when compared to the pre-exposure time point (**Figure 10A**).

#### Western Blot analysis for immunoreactivity to *P. duboscqi* salivary proteins

Western Blot results showed human volunteers’ plasma reactivity to *P. duboscqi* salivary proteins (~ 14-100kDa). Additionally, WB indicated some immunodominant proteins (~ 64, 44, 32, 20, 15kDa) which may be associated with the number of *P. duboscqi* exposures showing higher IgG reactivity in later sand fly exposures. These immunodominant proteins correspond to those detected in plasma from a naturally exposed Mali population used as positive control (**Figure 10B**). Furthermore, a more detailed analysis of additional western blot results (data not shown) indicated that the majority of the participants recognized same pattern of bands since exposure 2. The proteins with molecular weight (MW) around 15, 18, 52 and 84kDa are the ones most recognized by the majority of the participants at the different exposure times.

#### Cytokine production by PBMC stimulated with SGH and recombinant protein

**Figure 11:**
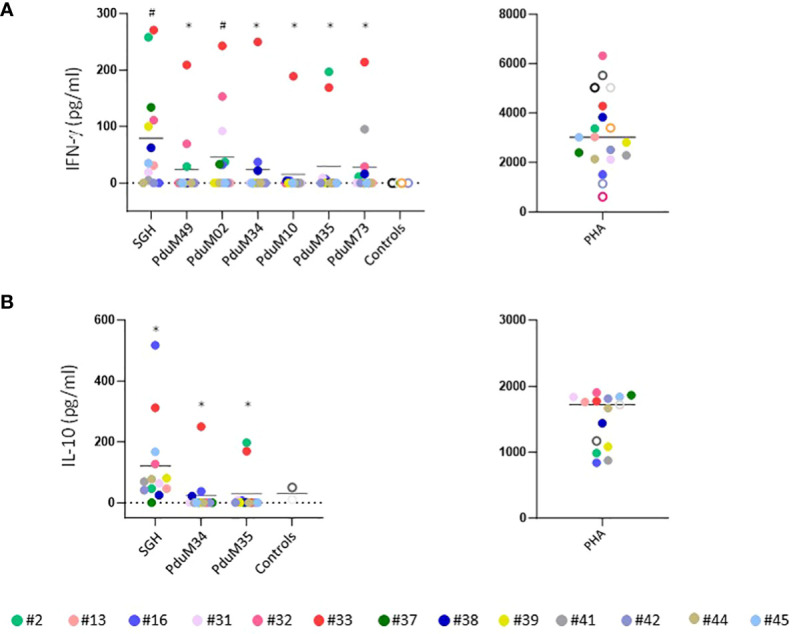
Characterization of the human cellular immune response to sand fly saliva or salivary recombinant proteins in volunteers experimentally exposed to *P. duboscqi* bites. **(A)** IFN-γ production (pg/ml) by Peripheral Blood Mononuclear cells (PBMCs) in response to a stimulation with SGH and recombinant salivary *P. duboscqi* proteins (PduM02, PduM10, PduM34, PduM35, PduM49 and PduM73) in exposed volunteers (n=13) versus blood bank donors (n=6). **(B)** IL-10 production (pg/ml) by Peripheral Blood Mononuclear cells (PBMCs) in response to stimulation with SGH and recombinant salivary *P. duboscqi* proteins (PduM02, PduM10, PduM34, PduM35, PduM49 and PduM73) in exposed volunteers (n=13) versus blood bank donors (n=2). Solid lines indicate the mean of each group. The * represents the statistical significance between SGH compared to the indicated recombinant protein. The # represents the statistical significance between stimuli versus controls.

Peripheral blood mononuclear cells (PBMC) collected at different exposure times from all volunteers were tested and compared to controls. Healthy donors from the National Institutes of Health blood bank were used as negative controls. Cellular immunity was assessed by measuring the IFN-γ and IL-10 production in the supernatants of PBMCs stimulated with SGH and with the six most abundant proteins from *P. duboscqi* saliva. These proteins are homologs to the small odorant proteins family (PduM02/SP15), to the silk related and collagen-like family (PduM34/SP32), the yellow family (PduM10/SP44 and PduM35/SP42), the adenosine deaminase-like protein (PduM73/adenosine deaminase) and a member of the SP15 family of proteins, PduM49/SP12 (20, 25). The PBMCs of the majority of volunteers (76%) responded specifically to stimulation with *P. duboscqi* SGH, producing IFN-γ in cell supernatants recovered 96 hours post stimulation (**Figure 11A**). All recombinant proteins induced some level of IFN-γ from PBMCs. However, PduM02/SP15 is the best candidate as it showed the highest response induced in 46% of volunteers. Also, 38.4% of volunteers responded specifically to stimulation with PduM73 (adenosine deaminase); 30% with PduM35 (Yellow SP42) and 23% with PduM49 (SP12), PduM34 (SP32) and PduM10 (Yellow SP44), producing IFN-γ (**Figure 11A**). One of the participants (#33) responded to all the recombinant proteins, where the others showed a more selective response.

The majority of volunteers (92%) responded to *P. duboscqi* SGH, producing IL-10 in cell supernatants recovered 96 hours post stimulation (**Figure 11B**). Regarding the low levels of IFN-γ produced after stimulation with the proteins PduM34 and PduM35, we observed a higher IL-10 induction in these samples where 61% of volunteers responded to stimulation with PduM34 (SP32) and 53% with PduM35 (Yellow SP42) (**Figure 11B**). Phytohemagglutinin (PHA) was used as positive control for the PBMC culture and was consistently elevated. Statistical differences were observed between cells stimulated with SGH when compared with all recombinant proteins in both IFN-γ (except for PduM02) and IL-10 stimulation. Statistical differences were observed in IFN-γ production between cells stimulated with SGH and PduM02 when compared to controls.

## Discussion

Vector challenge has been proposed in the development of leishmaniasis vaccines. Controlled human challenge models (CHIM), using infected sand fly bites, can provide a method for evaluation of prophylactic efficacy (26). CHIM studies, including the natural vector, may be particularly important when the process of vector transmission facilitates infection or alters immune responses in a manner not mimicked by needle challenge (27–29). Understanding the local effects associated with sand fly saliva may inform assessment of vaccine efficacy.

Individuals from endemic areas for leishmaniasis are frequently exposed to the bites of uninfected flies prior to bites of infected sand flies. Previous studies in an animal model showed that multiple exposures to uninfected sand fly bites induce specific immunity that can be detected by the presence of antibodies and cellular immune responses (16). Saliva-driven immunity protected against subsequent vector-transmitted leishmaniasis in mice, hamsters, and non-human primates (19, 20, 30). Little is known about the immune response resulting from interactions between humans and sand flies. In humans, the immunity post exposures to sand fly bites is dominated by a Th1, Th2 or a mixed immune response (31–33). Thus, an innovative approach could be the use of a Th1 immunogenic recombinant protein(s) from saliva of *P. duboscqi*, combined with parasite antigens, as a preventive vaccine.

The volunteer skin bite site reactions varied among our *P. duboscqi* exposed individuals. Erythema, induration and infrequent wheal and flare reactions were seen immediately post feeding, while papules, erythema, vesicles, reactivation of prior bite sites and macules were reported as delayed responses. Pruritus was a commonly reported symptom increasing in frequency over time and more common with delayed onset. Some volunteers recruited more cells into the bite site than others and the balance between the types of recruited cells vary. Indeed, Vinhas et al. (33), described that while three volunteers challenged with *Lutzomyia longipalpis* bites developed hemorrhagic points followed by small papules, the other three volunteers exhibited very mild early-phase reactions and later developed small nodular lesions. This corroborates with Abdeladhim et al. (3), described that not all individuals respond in the same way to sand fly salivary proteins and these differences may account for the various outcomes of cutaneous leishmaniasis in a population that is constantly bitten by sand flies. This difference is also demonstrated when we compared different timepoints of exposure, as seen in the skin biopsies of participants that presented a stronger immune response 2 months after prior exposure with both eosinophils and positive Luna staining when compared with 6 months recall participants, where one had no visible reaction and both only with mild mononuclear cell recruitment.

Vector-derived factors released during the sand fly bite can contribute to the inflammatory response with long-lasting effects on the host (3, 29, 34). In CL the amplification of eosinophil influx and their interaction with dermal Tissue-resident macrophages (TRM, cells responsible for maintaining tissue homeostasis) are associated with IL-4 stimulation, implicating eosinophil-TRM interactions in diverse inflammatory situations (35). Sanchez-Garcia et al. (36), demonstrated that in mice exposed to infected and non-infected *P. duboscqi* sand fly bites, mast cells increased in number continuously, reaching similar levels after 24 h. Moreover, at 48 h, bites of non-infected sand flies induced a higher number of neutrophils. Mast cells play a main role during inflammation after infection or tissue injury. They enhance the inflammatory process through paracrine regulation, where within minutes of degranulation, mast cells activate other cell types leading to intense inflammation (37). This massive release of granules containing potent inflammatory mediators may be related to the allergic effects observed in our study participants, suggesting that the immediate wheal and flare response noted in 3 volunteers could be triggered by mast cells.

Individuals naturally exposed to sand flies in endemic areas for leishmaniasis develop a humoral immune response to salivary molecules (30, 32, 38–42). Aronson, et al. (43), evaluating anti-sand fly saliva IgG levels of 248 persons with CL before and after Iraq deployment observed that level of antibody to *P. papatasi* salivary gland homogenate was significantly higher in exposed participants (after Iraq deployment) as compared to pre-deployment. Additionally, antibodies to saliva waned soon after leaving the endemic area, consistent with our participant #21 in this report, where anti-saliva IgG returned to baseline levels within two months of discontinued exposure. Previous studies demonstrated that individuals residing in a New World CL endemic area displayed antibody levels to *Lu. whitmani* salivary glands and that anti-saliva antibody levels were higher in CL patients compared to subclinical individuals (13).

Exposure to *Phlebotomus* species bites or salivary proteins favored a specific cellular and/or humoral immunity (24, 44, 45). Experimental exposure of naïve hosts to sand fly bites shows that antibody responses to saliva are acquired rapidly (33, 39, 46), increasing with the number of sand fly bites (46). Veysi et al. (47), exposed individuals to non-infected laboratory-bred *Phlebotomus* (*P. sergenti*) bites once a week for 8 months and found that at the beginning of *P. sergenti* bite exposure, IgG levels increased, however they noted a decreasing trend (seroreversion) by the end of the observation period. Our results showed that controlled exposure to uninfected *P. duboscqi* generated a humoral anti-saliva response with the levels of IgG increasing after each exposure. These results suggest that antibodies against sand fly saliva proteins may contribute as markers of vector exposure (48, 49) as potential surrogate markers for the risk of *Leishmania* infection (50) as well as a surveillance tool for monitoring vector control efforts after disease elimination (51).

In our study, exposed volunteers showed reactivity to *P. duboscqi* salivary proteins, although the intensity and composition of the immune reaction from the individuals was not uniform. Most participants recognized the majority of Western Blot bands by first assessment, after one month (two *P. duboscqi* exposure sessions). In our *P. duboscqi* study, some individuals recognized more intense bands around molecular weight 15, 18, 52 and 84 kDa after more *P. duboscqi* feeding sessions. As discussed by Barral et al. (38), immune reaction to sand fly salivary homogenate is complex and each antigen may elicit varying patterns of response in different individuals, and this may differ according to the timing and intensity of exposure. The diversity in the level of antibody and immunologic reactions in naturally acquired *P. sergenti* exposures was associated with individual specific antigenic electrophoretic patterns (52).

The selection of recombinant proteins used in the present work was based on the most abundant *P. duboscqi* salivary proteins, predicted from DNA plasmids injected into skin, that may be able to produce a protective cellular immune response (19). Kato et al. (25), investigating salivary proteins from two different sites (Mali and Kenya) discovered that at least five families of proteins (SP15-like, SP12-like, D7-like, antigen 5-like, and yellow-related protein) were 100% identical in sand flies collected from both countries. Interestingly, our data showed that participants present a specific Th1 immune response against not only SGH, but also to key recombinant salivary proteins from *P. duboscqi*. The majority of the volunteers produced IFN-γ against SP15. SP15 is known as homolog to the odorant-binding protein family and is found exclusively in the salivary glands of sand flies (53). Immunization with SP15 proteins induced IFN-γ and the development of type 1 immune response (18, 19).

Notably, around 40% of volunteers produced IFN-γ against adenosine deaminase (PduM73), which was previously identified in the sand fly *L. longipalpis* (54), *P. duboscqi* (53) and the mosquito *Aedes aegypti* (55), but not reported in other *Phlebotomus* species (25). Adenosine deaminase is an enzyme involved in the catabolism of purine bases and its main physiologic activity is related to lymphocytic proliferation and differentiation (56), suggesting an important role for immune responses and consequently as a potential target for leishmaniasis vaccine development. *P. duboscqi* transcripts coding for adenosine deaminase are responsible for the activity detected in the salivary glands of this sand fly and may be relevant for blood feeding, playing a role in parasite transmission (53). Adenosine is metabolized by adenosine deaminase to inosine, which has been described to result in a Th2 response in macrophages with decreased production of proinflammatory cytokines, somewhat contrary to the Th1 response we observed from adenosine deaminase (57).

We observed that SP32 and yellow protein/SP44 did not induce the production of high levels of IFN-γ, suggesting that specific proteins may stimulate the production of IL-10 instead. Indeed, Oliveira et al. (32), showed in leishmaniasis-endemic areas of Mali, PBMC from most individuals displayed a systemic immune response to sand fly saliva involving the production of Th1 and Th2 cytokines. Although 52% of individuals produced a mixed response, 23 and 25% displayed a Th1- or Th2-polarized response, respectively. Moreover, MW30 (presumably PpSP32) was the most frequently recognized antigenic salivary proteins present in 64.4% *L. major* CL cases transmitted in Iraq by the bite of a sand fly *P. papatasi* (43). Anti-saliva immune responses vary by individual and while contributory factors are incompletely understood, may be subject to multifactorial effects such as environment, season, other infections, nutritional status, and host genetic factors.

Our study has a few limitations including an inadvertent loss to follow-up after hospital closure and many volunteers had to move out of the area; fortunately, all had completed the intensive four biweekly exposures prior to that. In addition, *P. duboscqi* is the common vector for *L. major* in Sahel/sub-Saharan Africa, and our observations may not be generalizable more geographically broadly; however, *P. papatasi* saliva composition is similar to that of *P. duboscqi* and can be cross protective in mice, so this may not be a true limitation (58). One must consider the possibility of prior exposure to *Lutzomyia* species found in the US in our cohort as our screening was solely anti-saliva IgG which may be short lived. There are unique aspects to our report. Our study was the longest human challenge duration published using a controlled number of exposures with a high percentage of sand fly feeding, it investigated tissue level reactogenicity and provided a comparison between the bite site and the control arm skin biopsy, the participants were from a nonendemic area for phlebotomine sand flies, decreasing the possibility of having prior immunity against *P. duboscqi* saliva, and it used recombinant proteins to assess the systemic immune response to salivary proteins to describe immunogenic proteins that induce a Th1 immune response in humans.

## Conclusion

Our data showed the clinical and immunological consequences of *P. duboscq*i skin exposure over time. Sand fly bites resulted in both reactogenic and immunogenic skin responses with inter- and intra-participant variability in both the clinical reactions and systemic immune response. Humans showed specific cellular and humoral immune responses directed against sand fly salivary proteins and mainly to the recombinant proteins SP15 and adenosine deaminase inducing IFN-γ production, and SP32 and yellow protein/SP44 inducing IL-10 production. Our data reinforce the possible role of sand fly salivary molecules as components of a leishmaniasis vaccine. Future studies combining one or more recombinant vector salivary proteins with parasite antigen(s) for a more protective response constitute a novel leishmaniasis vaccine approach.

## Data availability statement

The raw data supporting the conclusions of this article will be made available by the authors, without undue reservation.

## Ethics statement

The studies involving humans were approved by Institutional Review Boards of the Walter Reed Army Medical Center, the Uniformed Services University of the Health Sciences, and the National Institute of Allergy and Infectious Diseases (NIAID), (Protocol number WR355023). The studies were conducted in accordance with the local legislation and institutional requirements. The participants provided their written informed consent to participate in this study. Written informed consent was obtained from the individual(s) for the publication of any potentially identifiable images or data included in this article.

## Author contributions

Fd: Conceptualization, Data curation, Formal analysis, Investigation, Methodology, Validation, Writing – original draft, Writing – review & editing. MA: Data curation, Investigation, Methodology, Writing – original draft, Writing – review & editing. CT: Data curation, Investigation, Methodology, Writing – review & editing. KH: Writing – review & editing, Project administration, Supervision. MW: Data curation, Formal analysis, Writing – original draft, Writing – review & editing. RR: Writing – review & editing, Data curation, Investigation, Supervision. IL: Data curation, Formal analysis, Investigation, Methodology, Writing – review & editing. ME: Writing – review & editing, Data curation, Investigation, Supervision. CM: Investigation, Methodology, Writing – review & editing. SN: Data curation, Methodology, Writing – review & editing. RG: Writing – review & editing, Investigation, Methodology. WT: Data curation, Methodology, Writing – original draft, Writing – review & editing. GT: Writing – review & editing, Data curation, Formal analysis, Investigation, Methodology. MP: Methodology, Supervision, Writing – original draft, Writing – review & editing. FO: Data curation, Investigation, Methodology, Writing – original draft, Writing – review & editing, Validation. JV: Conceptualization, Funding acquisition, Investigation, Resources, Supervision, Validation, Visualization, Writing – review & editing. SK: Conceptualization, Funding acquisition, Investigation, Resources, Supervision, Validation, Visualization, Writing – review & editing. NA: Conceptualization, Data curation, Funding acquisition, Investigation, Project administration, Resources, Supervision, Validation, Visualization, Writing – original draft, Writing – review & editing.

## Supplementary material

Supplementary Figure 1 Sand fly feeding throughout the scheduled exposures. Black bars represent sand flies with complete blood engorgement and white dashed bars represent sand flies with partial feeding.
