# Supplementary figures and images for: Immune response profiles from humans experimentally exposed to Phlebotomus duboscqi bites
Source: Front Immunol. 2024 Apr 3;15:1335307. doi: 10.3389/fimmu.2024.1335307 (PMC11021656; doi:10.3389/fimmu.2024.1335307)

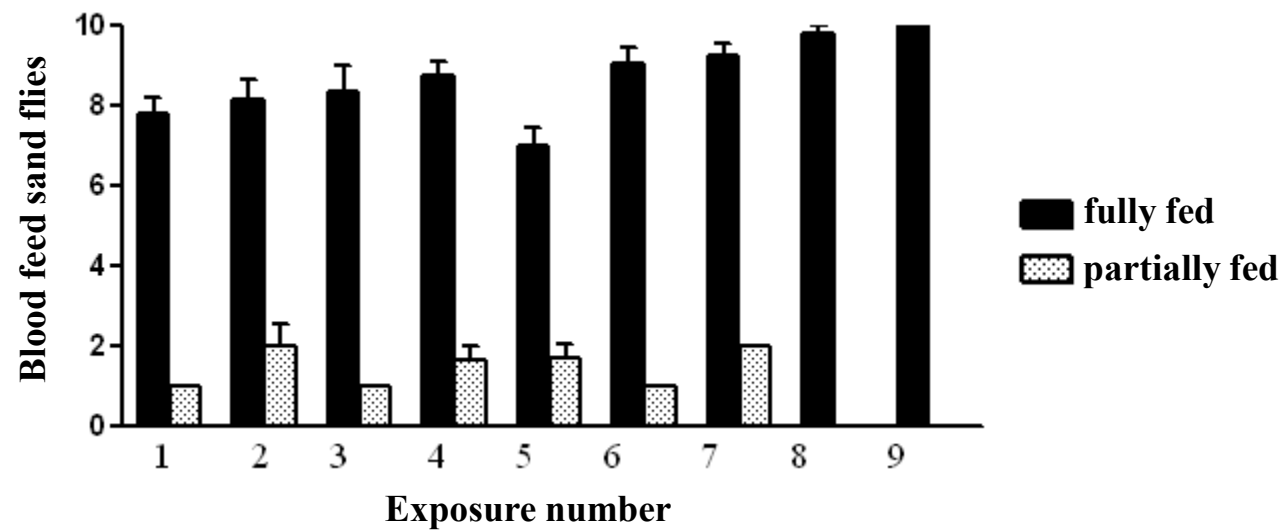

Supplement: Supplementary Figure 1 — Sand fly feeding throughout the scheduled exposures. Black bars represent sand flies with complete blood engorgement and white dashed bars represent sand flies with partial feeding. [file DataSheet_1.pdf]
